# ChatGPT in medicine: correspondence

**DOI:** 10.1097/JS9.0000000000001947

**Published:** 2024-07-08

**Authors:** Hinpetch Daungsupawong, Viroj Wiwanitkit

**Affiliations:** a Private Academic Consultant, Phonhong, Lao People’s Democratic Republic; b Department of Research Analytics, Saveetha Dental College and Hospitals, Saveetha Institute of Medical and Technical Sciences Saveetha University, Chennai, Tamil Nadu, India

*Dear Editor*,

We would like to discuss ‘A new ChatGPT-empowered, easy-to-use machine learning paradigm for environmental science’^1^. Researchers have looked into the uses and difficulties of ChatGPT, an OpenAI generative AI chatbot, in a variety of medical contexts in the digital age of medicine. The latest application of artificial intelligence by offering precise, individualized, and current health information to both tourists and medical professionals, ChatGPT has the potential to completely transform the field of travel medicine^2^. ChatGPT can assist travelers in planning ahead and maintaining their health while on the road by utilizing AI and NLP technology^2^. While ChatGPT might be a useful tool for teaching, it cannot take the place of in-person instruction. Dissection-based laboratory sessions are still necessary for students to gain practical skills and spatial awareness in veterinary anatomy education^3^. The article emphasized ChatGPT’s possible applications in patient care, clinical evaluation, research, and teaching. However, the study missed crucial topics, including medication interactions, drug development, and reducing racial and ethnic inequities, which raises questions about how thorough the conversation was.

The lack of emphasis on medication interactions, where ChatGPT performed inconsistently in terms of accuracy and specificity when compared to other AI chatbots, is one of the article’s weaknesses. Despite its ability to accelerate the process, ChatGPT’s potential for drug discovery and development has not been fully explored. Potential drawbacks to the technology’s application were also noted, including concerns about depending too heavily on ChatGPT responses and whether they are appropriate for public questions.

Subsequent research may look into ChatGPT’s ability to evaluate pharmaceutical interactions, improve drug development procedures, and reduce racial and ethnic disparities in medical data analysis. More research may be interested in examining ChatGPT’s capacity to manage multimodal data, fight disinformation, and improve transparency in scientific publications. Furthermore, in order to guarantee dependable and trustworthy results for patients and healthcare professionals, it will be essential to validate the accuracy and comprehensiveness of ChatGPT’s responses in medical settings.

All things considered, ChatGPT offers interesting chances for healthcare innovation, but it also has drawbacks in terms of accuracy, dependability, and moral issues. Subsequent studies should aim to address these limitations by conducting rigorous studies of ChatGPT’s performance in other medical domains, such as pharmaceutical interactions, drug discovery, and racial and ethnic differences. To fully realize AI’s potential in enhancing patient care and furthering medical research, more research and development in this field will be necessary.

## Ethical approval

Not applicable.

## Consent

Not applicable.

## Source of funding

Not applicable.

## Author contribution

H.P.: 50 % ideas, writing, analyzing, and approval; V.W.: 50 % ideas, supervision, and approval.

## Conflicts of interest disclosure

The authors declare no conflicts of interest.

## Guarantor

Professor Viroj Wiwanitkit.
